# On Gangrenous Erosion in Children

**Published:** 1827-06

**Authors:** Edward Thompson

**Affiliations:** Member of the Royal College of Surgeons, &c.


### DISEASES OF CHILDREN.

### *On Gangrenous Erosion in Children.* By Edward Thompson, Esq. Member of the Royal College of Surgeons, &c.

There is an affection, with which children more particularly are attacked, which in the majority of cases proceeds to a fatal termination, independent of the various and active treatment that has at different times been recommended and resorted to. It makes its seizure in different ways, the part being attacked with gangrene at once, or spreading by gangrenous ulceration,—being either preceded or not by a fever of a low type. By Vogel it has been named Noma; and by different other writers designated by the terms Gangrenous Excoriation, or Erosion. Some have attempted to draw a line between the terms, and have wished to make distinct diseases of but one affection; but the grounds of difference are not strong, and it is more than probable that they are modifications only of one disease.

It makes its attack generally in weakly, ill fed and ill clothed children; and is a consequence of debilitating complaints, occasionally, in those of better condition. I have observed that it commences more frequently at the junction of the skin and mucous membrane of one part or other of the lip, and spreads on both sides, with equal rapidity, to parts in the neighbourhood. The action is inflammatory, ending almost immediately in mortification, and this with such quickness that sometimes the cheek becomes entirely destroyed in a few hours. The disease spreads by continuity; for, at the distance of a line or two, nothing particular can be observed in the part so soon to be destroyed. By whatever cause the disease is excited, when it has once begun, the state of the habit contributes to its increase, and, although arising from circumstances perfectly local, yet it must be looked upon as having to do with the constitution at large; a state of the system being present which adds strength to the disorder, being possessed of little or none of the power which a healthy body has of checking a process destructive to life.

The causes of the complaint are not known, but there is reason to think that the disease is of local origin. I do not know that fever generally precedes it; but, after it has been established, fever is a common attendant, apparently excited by the state of irritation which, as the affection increases, becomes excessive.

In the treatment of this formidable disease, medical men have occasionally differed; but the farther they have gone into refined speculations respecting the constitutional nature of the complaint, and left unheeded the local disorder, the want of success has been more evident. No one that has seen the disease, and treated it, would depend on constitutional means, unaided by local remedies. In Mr. DEASE's cases, strict attention was paid to the state of local action, and his success is without parallel. But, with all kinds of treatment, the affection is one as dangerous in its character as any with which the practitioner has to cope, and oftentimes all his resources will avail him nothing, failure accompanying every effort.

The object of this short paper is to recommend a simple remedy, which has in my hands proved useful, and may therefore do so in others. It is one which has, in cases of sloughing ulcer, shown peculiar power, and was a few years ago recommended as a remedy in hospital gangrene. I mean the Balsam of Peru. Its efficacy in this affection induced me to employ it in gangrenous erosion of the lip and cheek; and, to prove that in this dangerous complaint it may be used with advantage, I beg leave to make a short abstract from my note-book of a case that occurred to me some time back, as threatening in its appearance as any I have seen.

On Thursday, the 1st of April, 1824, George Hadwin, a delicate boy, aged three years, complained of an uneasy feeling in the lip. When examined, a small hard spot was observed, slightly discoloured. The day after, this spot was found in a state of complete mortification, and beginning to separate. During the day it came away. After this the sloughing became more extensive and rapid, so as to destroy nearly the whole of the upper lip, and part of the cheek, in the short space of two days.

On Monday, the 5th of April, when I was desired to see him, the part had a most horrid appearance; several of the teeth hanging loose in the mouth, the gangrene having extended to the gums in the upper and lower jaws. The part was ragged, and of great extent. Round the ulcer was a narrow line of inflammation; but beyond this pencil of inflammation, which was not more than half a line in breadth, the integument had a natural appearance. The child was weak, with a haggard and pallid countenance. Sleep had completely left it; and to all appearance it was in a state of great danger. At such times, when so much is required, the mind turns to a variety of means, without determining which to fix upon, as all confidence in any of them having the power to arrest a malady known to be so fatal is lost, and one remedy seems nearly as important as another. In such a state of uncertainty, the following were directed, with little expectation of benefit.

Parti affectæ applic$^{r}$ Bals. Peruvianum.—℞. Mist. Camphoræ ℥ iij.; Pulv. Cinchonæ ʒ iij.; Tinct. Opii gtt. xij. M. cujus capiat cochlear. demidium secundâ quâque horâ.

6th.—A little better; sloughing apparently stopped; redness round the edges abated, and the sore assuming a florid hue. No appetite; bowels relaxed.

To have port-wine and water as drink. Diet to be formed of animal jellies and vegetable mucilages.—Contin$^{r}$ med.

7th.—Sore looking better; gangrenous inflammation diminished, and sloughing ceased. Slept a little in the night. Appetite improving.

8th.—Still improving. Sore healthy, except that the edges of the ulcer are slightly hard.

Sumat Ext. Hyosciami gr. jss. quartâ quâque horâ.

9th.—In every respect better. Several teeth in the upper jaw removed, being quite loose and unsupported by the gum.

Contin$^{r}$ medicamenta et alia.

10th.—Part gradually improving.

Pergat.

11th.—Sore rapidly healing.

On the 12th, I ceased to visit him, the place being nearly healed, and his health established.

I had an opportunity of seeing this boy about twelve months ago, and was much surprised to find that nature had done so much in restoring what disease had so hastily removed. In the process of healing, the contraction of the skin had caused an erosion of the mucous membrane of the mouth, to such an extent that the deformity appeared as nothing when compared to what it was when I attended him. I have read in some continental publication, that RICHERAND, depending on such a renovating process, has removed the whole of the lip in cases of cancer, and with decided success; a regeneration taking place in a singular manner. From what has been done in this case by nature alone, (for a reunion by a surgical operation was out of the question,) I should have little doubt of such a process following most cases of loss of substance, either in the cheek or lip.

In the affection called cancrum oris, a disease of more common occurrence than the one above spoken of, but which possesses many of its characters, Dr. Coats has found a solution of sulphate of copper the most useful application. This is an old remedy, and was formerly more used than at present: with Dr. Parr it was a favourite one; but in erosion of the cheek, I can only say I have not found it to possess any power. It was employed in Hadwin's case, before I saw the child, but with no effect, the sloughing continuing without abatement.

On the peculiar way in which balsam of Peru acts in affections of this nature, little can be said. The pathology of the disease is hid in obscurity, and, till sounder principles are established, our means must partake not a little of an empirical character. The first state of the part attacked is that of inflammation; but whether the nervous energy is so far reduced that, almost immediately after such action has been formed, death of the part follows, from want of power to continue it, or the disease lies more particularly in the vessels, cannot be determined. But, at all events, a warm stimulant appears to check the progress of the mischief, and I know of none better suited for such purpose than Peruvian balsam.

The way it has been used has been by keeping the slough, and parts around, constantly soaked with warm balsam, smeared on lint; occasionally removing the sloughs, avoiding by every possible means the exposure of the surface to the action of the air. Attention to this is of paramount importance; for it is a fact, although difficult to explain, that the gangrenous inflammation proceeds more rapidly, if such precaution be neglected.

I may mention, before concluding, that it has been proposed to remove the diseased part with the knife, beyond the mark of inflammation; yet this operation, as having a tendency to excite inflammation, cannot be expected to be productive of benefit. Caustic has been employed in another complaint of a somewhat similar nature, and it is stated with some success. The actual cautery is another mean that has occasionally been tried; but all these have, in the greater number of cases, shown a tendency to extend the disease which they were employed to check: such being the case, their curative powers, in the complaint more immediately under consideration, is at best but doubtful.

*Whitehaven; April* 1827.

